# CircTUBGCP3 facilitates the tumorigenesis of lung adenocarcinoma by sponging miR-885-3p

**DOI:** 10.1186/s12935-021-02356-2

**Published:** 2021-12-06

**Authors:** Yang Yang, Xin Fan, Yunfei Nie, Donglei Liu, Dengyan Zhu, Kai Wu, Yuan Zhang, Wenhua Li, Xiangyu Tian, Huaqi Wang, Yuxia Fan

**Affiliations:** 1 Department of Thoracic Surgery, The First Affiliated Hospital of Zhengzhou University, Zhengzhou, China; 2 Department of Urology, The First Affiliated Hospital of Zhengzhou University, Zhengzhou, China; 3 Department of Orthopedics, The First Affiliated Hospital of Zhengzhou University, Zhengzhou, China; 4 Department of Respiratory, The First Affiliated Hospital of Zhengzhou University, No. 1 Jianshe East Road, Erqi District, 450052 Zhengzhou, China; 5 Department of Thyroid Surgery, The First Affiliated Hospital of Zhengzhou University, No. 1 Jianshe East Road, Erqi District, 450052 Zhengzhou, China

**Keywords:** circTUBGCP3, miR-885-3p, Wnt10b, Lung adenocarcinoma, Growth

## Abstract

**Background:**

Circular RNAs (circRNAs) act pivotal roles in the progression of multiple malignancies. However, the underlying mechanisms by which hsa_circ_0007031 (circTUBGCP3) contributes to lung adenocarcinoma (LAC) remain largely unknown.

**Methods:**

The association of circTUBGCP3 expression with clinicopathological characteristics and prognosis in patients with LAC was determined by RT-qPCR and fluorescence in situ hybridization. The in vitro functional experiments as well as a subcutaneous tumorigenesis model were executed to estimate the role of circTUBGCP3 in LAC cells. The interaction between circTUBGCP3 and miR-885-3p was confirmed by RNA immunoprecipitation, luciferase gene report and RT-qPCR assays. The effects of circTUBGCP3 on miR-885-3p-mediated Wnt10b/β-catenin signaling were evaluated by Western blot.

**Results:**

The upregulation of circTUBGCP3 or downregulation of miR-885-3p was associated with the pathological stage and poor survival in patients with LAC. Restored expression of circTUBGCP3 facilitated the growth and invasion of LAC cells, but knockdown of circTUBGCP3 harbored the opposite effects. In mechanism, circTUBGCP3 could act as a sponge of miR-885-3p, which suppressed the cell proliferation and colony formation and attenuated the tumor-promoting effects of circTUBGCP3. Wnt10b as a target of miR-885-3p could be upregulated be circTUBGCP3 and indicate poor survival in patient with LAC.

**Conclusions:**

Our findings demonstrated that circTUBGCP3 promoted LAC progression by sponging miR-885-3p, and might represent a prognostic factor for LAC.

**Supplementary Information:**

The online version contains supplementary material available at 10.1186/s12935-021-02356-2.

## Introduction

Lung cancer harbors the high incidence rate and mortality worldwide, and non-small cell lung cancer (NSCLC) accounts for 85% of the total cases in lung cancer [1]. Although extensive progress has been made to improve the outcomes of NSCLC, the therapeutic effect is unsatisfactory duo to the tumor invasiveness and metastasis [2]. Increasing data indicate that the aberrant expression of noncoding RNAs (ncRNAs) is implicated in the progression of NSCLC [3, 4]. Identification of underlying ncRNAs may offer insights into the detection of NSCLC.

Circular RNAs (circRNAs) as a novel subgroup of ncRNAs are characterized by a closed loop structure, tissue-specific expression and resistance to RNase R [5]. It has been reported that circRNAs can sponge miRNAs, bind with protein and favor protein translation in cancers. CircFOXP1 interacts with PTBP1 to accelerate the Warburg effect in gallbladder cancer [6], circMYBL2 modulates FLT3 translation by recruiting PTBP1 in AML [7] and circDLST sponges miR-502-5p to enhance the metastasis of gastric cancer [8]. Moreover, circRNAs act a critical role in LAC. CircSLC25A16 [9], circSATB2 [10] and circNT5E [11] contribute to glycolysis and cell invasion in LAC by sponging miR-326/-134. Downregulation of circPTPRA [12] or upregulation of circHIPK3 [13] harbors an association with poor prognosis in LAC.

It has been reported that hsa_circ_0007031 can promote the tumorigenesis of osteosarcoma by sponging miR-30b [14]. Herein, we identified a differentially-expressed hsa_circ_0007031 (circTUBGCP3) between LAC and adjacent normal tissues, and found that upregulation of circTUBGCP3 or downregulation of miR-885-3p was associated with pathological stage and poor survival in LAC. CircTUBGCP3 promoted the tumorigenesis of LAC by sponging miR-885-3p and indicated poor prognosis in LAC.

## Materials and methods

### Clinical data

The clinical data for LAC tissues as well as the relative expression levels of EIF4A3, Wnt10b and miR-885-3p were downloaded from The Cancer Genome Atlas (TCGA) RNA-seq database (https://genome-cancer.ucsc.edu). 8 paired LAC biopsy tissues were preserved in liquid nitrogen in our hospital. A tissue microarray including 90 paired LAC tissues was purchased from Superbiotek Pharmaceutical Technology (Shanghai, China). The specimens were classified according to the TNM staging, and diagnosed by two independent pathologists. Our study was approved by the Ethics Committee of Zhengzhou University.

### CircRNA expression profiling

Total RNA from LAC and adjacent normal tissues (n = 3) was quantified using the NanoDrop ND-1000. The sample preparation and microarray hybridization were conducted according to the Arraystar’s standard protocols. The detailed description of this process was carried out according to the previous report [15].

### Identification of circTUBGCP3-specific binding with miRNAs

CircTUBGCP3 specific binding with miRNAs (miR-885-3p, miR-640, miR-324-5p, miR-194-3p and miR-103a-3p) was identified by circRNA expression profiling and miRbase. The targets of miR-885-3p were identified by TargetScan7.1 (http://www.targetscan.org/vert_71/).

### Cell culture

Normal pulmonary epithelial cells BEAS-2B and LAC cell lines (A549, NCI-H23, NCI-H1993, SPC-A1, NCI-H460) were stored in liquid nitrogen in our hospital. They were cultured in Dulbecco’s Modified Eagle medium (DMEM) medium supplemented with 10% heat-inactivated fetal bovine serum (FBS), 100 U/ml of penicillin, and 100 µg/ml of streptomycin (HyClone) in a humidified atmosphere containing 5% CO_2_ at 37℃.

### Fluorescence in situ hybridization (FISH)

Digoxin-labeled probe sequences for circTUBGCP3 (5’-GGATCACATCATTGC TGCAC-3’) were used for analysis of the expression and localization of circTUBGCP3 in LAC tissues and cells. The detailed description of FISH analysis was conducted as previously reported [8]. The analysis software Image-pro plus 6.0 (Media Cybernetics, Inc., Rockville, MD, USA) was used to obtain the immunofluorescence accumulation optical density of circTUBGCP3 in LAC tissues.

### Quantitative real-time PCR (qRT-PCR)

Total RNA was extracted by using TRIzol, reverse transcription was performed by using M-MLV and cDNA amplification by using the SYBR Green Master Mix kit (Takara, Otsu, Japan). Total RNA was isolated using a High Pure miRNA isolation kit (Roche) and RT-PCR using a TaqMan MicroRNA Reverse Transcription kit (Life Technologies). The nuclear and cytoplasmic fractions were isolated using NE-PER Nuclear and Cytoplasmic Extraction Reagents (Thermo Scientific). The primers were listed in Additional file 1: Table S1.

### Western blot analysis

LAC cell lines (NCI-H460 and A549) were harvested and extracted by using lysis buffer. Cell extracts were boiled in loading buffer and equal amount of cell extracts were separated on 15% SDS-PAGE gels. Separated protein bands were transferred into polyvinylidene fluoride membranes. The primary antibodies anti-Wnt10B (ab70816, Abcam, USA), anti-β-catenin (ab2365, Abcam, USA) and anti-GAPDH (#5174, CST, Shanghai, China) were diluted at a ratio of 1:1000 according to the instructions and incubated overnight at 4℃. The detailed description of Western blot analysis was performed as previously reported [8].

### Plasmid, siRNA, miRNA mimic and inhibitor

Plasmid-mediated circTUBGCP3 vectors, lentivirus-mediated siRNA targeting circTUBGCP3 vector (si-circTUBGCP3, 5’-GACAGTGACTCCAGGTTTTTT-3’) and miR-885-3p mimic/inhibitor and EIF4A3 plasmids were purchased from GenePharma (Shanghai, China). The negative controls such as NC, si-NC, pEX-3 or miR-NC was used as the control vectors. NCI-H460, A549 and SPC-A1 cell lines were planted in 6-well plates 24 h prior to si-circTUBGCP3, circTUBGCP3, miR-885-3p mimic or inhibitor transfection with 50-60% confluence, and then were transfected with Lipofectamine 2000 (Invitrogen, Carlsbad, CA, USA) according to the manufacture instructions.

### Luciferase reporter assay

NCI-H460, A549 and SPC-A1 cell lines were seeded into 96-well plates and were co-transfected with PRL-TK-pMIR-circTUBGCP3 or PRL-TK-pMIR-Wnt10b 3’UTR, and miR-885-3p mimic/inhibitor or miR-NC. After 48 h of incubation, the firefly and Renilla luciferase activities were detected with a dual-luciferase reporter assay (Promega, Madison, WI, USA).

### MTT and Transwell assays

MTT and Transwell assays were performed as previously reported [15].

### Colony formation assay

A549, SPC-A1, NCI-H460 and NCI-H1299 cells were seeded into 6-well plates (2 × 10^3^ cells per well) and treated with sh-circTUBGCP3 or circTUBGCP3 plasmids after the cells were attached to the wall, culturing for 7 days. Cells were fixed with 4% paraformaldehyde and stained with 0.1% crystal violet for 30 min. Colony formation assay was examined by counting the number of stained colonies.

### RNase R and Actinomycin D treatment

Total RNA (2 µg) was incubated for 30 min at 37℃with 3 U/µg of RNase R (Epicentre Technologies, Madison, WI, USA). Transcription was prevented by the addition of 2 mg/ml Actinomycin D or DMSO (Sigma-Aldrich, St. Louis, MO, USA) as the negative control. After A549 and SPC-A1 cell lines were exposed to RNase R or Actinomycin D treatment, the enrichment levels of circTUBGCP3 and TUBGCP3 were detected by qPCR analysis.

### RNA immunoprecipitation (RIP)

RIP assay was performed in A549 and SPC-A1 cell lines by using a Magna RIP RNA-binding protein Immunoprecipitation Kit (Millipore) according to the manufacturer’s instructions. Antibodies for RIP assays against Ago2 (ab5072) and IgG were purchased from Abcam, USA.

### In vivo tumorigenesis assay

Male nude mice (6 weeks old) were purchased from Shanghai SIPPR-BK Laboratory Animal Co. Ltd (Shanghai, China) and maintained in microisolator cages. All the animals were conducted according to the institutional guidelines, and approved by the Animal Ethics Committee of Zhengzhou University. The mice were subcutaneously inoculated with 1 × 10^7^ of NCI-H460 cells stably transfected with si-circTUBGCP3 or si-NC. The mice were killed by cervical dislocation. After that, the body weight and tumor size were measured every other day, and the tumor volume was obtained according to the formula: length × width^2^/2.

### Statistical analysis

Statistical analyses were conducted by using SPSS 20.0 (IBM, SPSS, Chicago, IL, USA) and GraphPad Prism. Student’s t-test or Chi-square test was used to analyze the statistical data between two groups, but Analysis of Variance was used to estimate the statistical significance for comparisons of more than three groups. Overall survival curves were analyzed with the Kaplan-Meier method and log-rank test. Univariate analysis and multivariate models were performed by using a Cox proportional hazards regression model. *P* < 0.05 was considered statistically significant.

## Results

### Upregulation of circTUBGCP3 was associated with poor survival in LAC

**Fig. 1:**
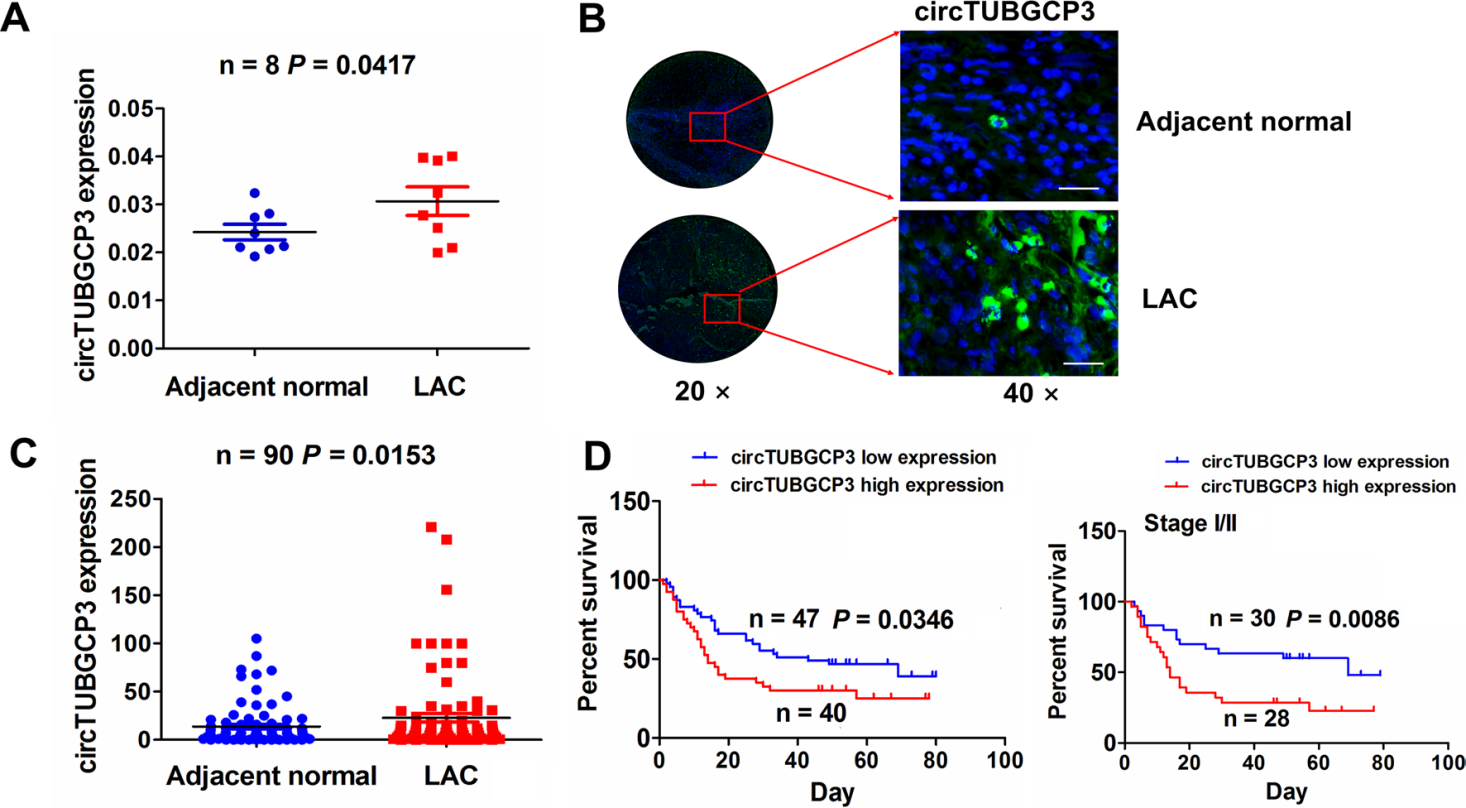
The upregulation of circTUBGCP3 was associated with poor survival in LAC patients. **A** qPCR analysis of the expression level of circTUBGCP3 in LAC tissues. **B**, **C** FISH validation of the expression level of circTUBGCP3 in 90 paired LAC tissues. Blue color for DAPI staining represented the cell nucleus, and green color for circTUBGCP3 probe. **D** Kaplan Meier analysis of the association of high or low circTUBGCP3 expression with overall survival in LAC as well as early stage cases

According to the fold change (FC > 2) and *p* value (< 0.02), the circRNA profiling was used to identify the differentially-expressed circRNAs between LAC and normal tissues. We found that hsa_circ_0007031 expression level harbored the most obvious increase in LAC tissues (FC = 28.101, *p* < 0.0001; Additional file 1 Fig. S1A). According to the circRNA interactome, hsa_circ_0007031 (chr13:113158345-113181798) is originated from exon 11, 20 regions within tubulin gamma complex associated protein 3 (TUBGCP3) locus and named as circTUBGCP3, which is located on chromosome 13q34 and its spliced length is 972nt (Additional file 1: Fig. S1B). The expression level of circTUBGCP3 was identified to be elevated in LAC tissues by RT-qPCR analysis (Fig. 1A). This result was further validated by FISH analysis (Fig. 1B, C).

A cutoff value of circTUBGCP3 was gained and divided the cases into high-circTUBGCP3 and low-circTUBGCP3 groups. We found that elevated expression of circTUBGCP3 was associated with age (*P* = 0.030) and pathological stage (*P* < 0.0001, Table 1). The cases with high-circTUBGCP3 level possessed a worse survival as compared with those with low-circTUBGCP3 level, and the similar result was indicated in early-stage cases (Fig. 1D). However, the late-stage patients with high-circTUBGCP3 level had no difference in overall survival as compared with those with low-circTUBGCP3 level (Additional file 1: Fig. S1C). Univariate and multivariate analyses unveiled circTUBGCP3 as an independent factor of poor survival in LAC (Table 2).

**Table 1:** The association of circTUBGCP3 expression with clinicopathological characteristics in LAC patients

Variables	Cases (n)	circTUBGCP3		*P* value
		High	Low	
Total	87	40	47	
Age (years)				
≥60	21	14	7	
<60	66	26	40	0.030
Gender				
Male	78	38	40	
Female	9	2	7	0.133
Pathological stage				
I/II	58	36	22	
III/IV	29	4	25	<0.0001
T stage				
T1/T2	46	23	23	
T3/T4	41	17	24	0.428

**Table 2:** Cox regression analysis of circTUBGCP3 expression as a survival predictor

Variables	Univariate Cox regression analysis		Multivariate Cox regression analysis	
	RR (95% CI)	*P* value	RR (95% CI)	*P* value
Age (years)				
≥60 vs. <60	1.051 (0.572 to 1.930)	0.872	NA	NA
Gender				
Male vs. Female	1.942 (0.701 to 5.379)	0.202	NA	NA
Pathological stage				
III/IV vs. I/II	0.882 (0.498 to 1.563)	0.666	0.874 (0.487 to 1.569)	0.651
TNM stage				
T3+T4 vs. T1+T2	1.207 (0.711 to 2.050	0.486	1.319 (0.766 to 2.271)	0.318
circ_0007031 expression				
High vs. Low	1.752 (1.028 to 2.984)	0.039	1.787 (1.044 to 3.059)	0.034

*NA* not analyzed

### CircTUBGCP3 was identified as a circRNA in LAC cells

**Fig. 2:**
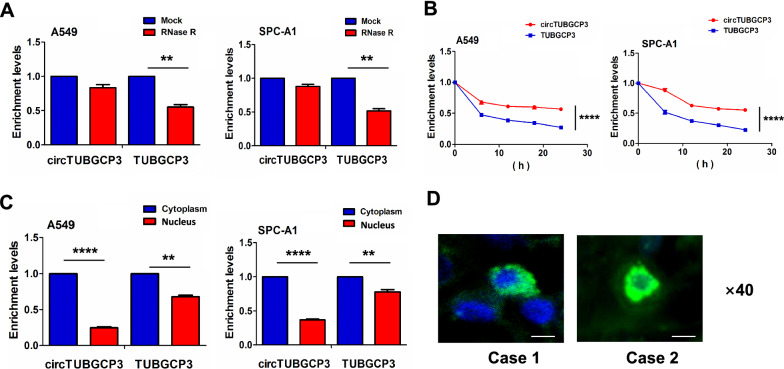
Identification of circTUBGCP3 as a circRNA in LAC cells. **A** qPCR analysis of the enrichment levels of circTUBGCP3 and TUBGCP3 after the A549 and SPC-A1 cells were exposed to the treatment of RNase R. **B** qPCR analysis of the enrichment levels of circTUBGCP3 and TUBGCP3 after the A549 and SPC-A1 cells were exposed to Actinomycin D at indicated time points. **C** qPCR analysis of the cellular localization of circTUBGCP3 and TUBGCP3 in A549 and SPC-A1 cells. **D** FISH analysis of the cellular localization of circTUBGCP3 in LAC tissue cells. Blue color for DAPI staining represented the cell nucleus, and green color for circTUBGCP3 probe represented the cell cytoplasm in LAC tissues. Data are the means ± SEM of three experiments. **P < 0.01; ****P < 0.0001

According to RT-qPCR results, circTUBGCP3 produced a resistance to the digestion of RNase R as compared with the linear TUBGCP3 in A549 and SPC-A1 cell lines (Fig. 2A). These two cell lines were exposed to the transcription inhibitor Actinomycin D at indicated time points. The RT-qPCR analysis showed that the half-life of circTUBGCP3 exceeded 24 h, while that of linear TUBGCP3 lasted for 6 h (Fig. 2B). RT-qPCR (Fig. 2C) and FISH analysis (Fig. 2D) demonstrated that circTUBGCP3 was predominantly localized in the cytoplasm of LAC tissue cells.

### CircTUBGCP3 facilitated the growth and invasion of LAC cells

**Fig. 3:**
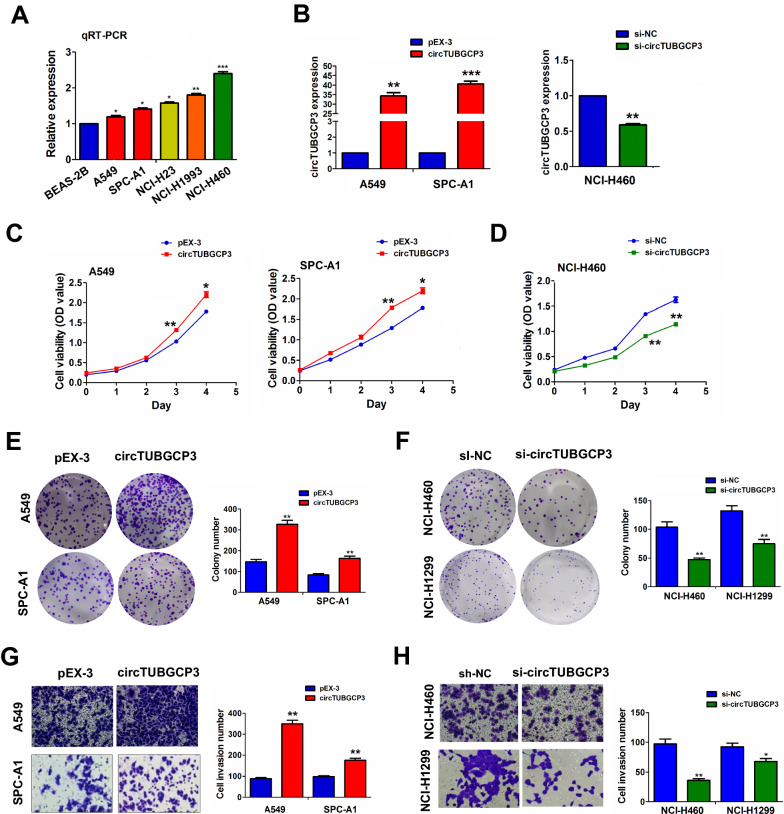
Restored expression of circTUBGCP3 promoted the proliferation, colony formation and invasion of LAC cells. **A** qPCR analysis of the expression levels of circTUBGCP3 in multiple LAC cell lines. **B** qPCR analysis of the efficiency of plasmids-mediated circTUBGCP3 in A549 and SPC-A1 cell lines or lentivirus-mediated si-circTUBGCP3 in NCI-H460 cell line. **C**, **D** MTT analysis of the cell viability after the transfection with circTUBGCP3 plasmids in A549 and SPC-A1 cell lines or si-circTUBGCP3 in NCI-H460 cell line. **E**, **F** Colony formation analysis of the colony formation capabilities after the transfection with circTUBGCP3 plasmids in A549 and SPC-A1 cell lines or si-circTUBGCP3 in NCI-H460 and NCI-H1299 cell lines. **G**, **H** Transwell analysis of the cell invasive potential after the transfection with circTUBGCP3 plasmids in A549 and SPC-A1 cell lines or si-circTUBGCP3 in NCI-H460 and NCI-H1299 cell line. Data are the means ± SEM of three experiments. *P < 0.05, **P < 0.01, ***P < 0.001

The expression levels of circTUBGCP3 were measured in multiple cell lines by RT-qPCR analysis, which indicated that circTUBGCP3 harbored an elevated expression level in A549 and SPC-A1 cell lines and possessed relatively higher expression levels in NCI-H460 and NCI cell lines as compared with normal pulmonary epithelial cell line BEAS-2B (Fig. 3A). The efficiencies of plasmids-mediated circTUBGCP3 in A549 and SPC-A1 cells or the interference efficiencies of lentiviruses-mediated si-circTUBGCP3 in NCI-H460 cells were respectively examined by RT-qPCR analysis (Fig. 3B). Further investigations indicated that restored expression of circTUBGCP3 accelerated the cell viability in A549 and SPC-A1 cells (Fig. 3C),while knockdown of circTUBGCP3 repressed the cell viability in NCI-H460 cells (Fig. 3D). Likewise, overexpression of circTUBGCP3 promoted the cell colony formation in A549 and SPC-A1 cells (Fig. 3E), whereas circTUBGCP3 knockdown reversed these effect in NCI-H460 and NCI-H1299 cells (Fig. 3F), The similar results were indicated by Transwell assay in Fig. 3G, H.

### CircTUBGCP3 acted as a sponge of mR-885-3p in LAC cells

**Fig. 4:**
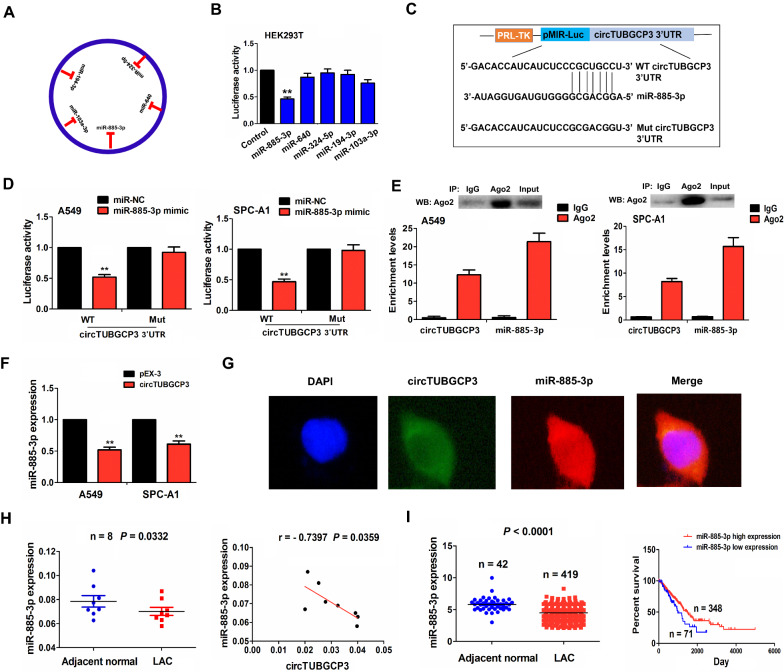
CircTUBGCP3 acted as a sponge of mR-885-3p in LAC cells. **A** Identification of circTUBGCP3 specific binding with 5 miRNAs. **B** The luciferase activity of circTUBGCP3 3’UTR after the treatment with 5 miRNAs in HEK293T. **C** The binding sites between WT or Mut circTUBGCP3 3’UTR and miR-885-3p. **D** The luciferase activity of circTUBGCP3 3’UTR after the treatment with miR-885-3p mimic in A549 and SPC-A1. **E** RIP assay analysis of the binding between circTUBGCP3 or miR-885-3p and Ago2 protein in A549 and SPC-A1. **F** qPCR analysis of the expression levels of miR-885-3p after the transfection with circTUBGCP3 plasmids in A549 and SPC-A1. **G** FISH analysis of the co-localization of circTUBGCP3 with miR-885-3p in A549. **H** qPCR analysis indicated the downregulation of miR-885-3p and its negative correlation with circTUBGCP3 in LAC tissues. **I** TCGA cohort validated the downregulation of miR-885-3p in 419 LAC tissues and Kaplan Meier analysis of the association of high or low miR-885-3p expression with overall survival in LAC patients. Data are the means ± SEM of three experiments. **P < 0.01

According to the circRNA profiling and miRbase, 5 miRNAs (mR-885-3p, mR-640, mR-324-5p, mR-194-3p, mR-103a-3p) were identified to have the potential to bind with circTUBGCP3 (Fig. 4A, Additional file 1: Fig. S2). Luciferase report indicated that the luciferase activities of circTUBGCP3 3’UTR were markedly reduced by mR-885-3p mimic rather than other miRNAs as compared with the control group in HEK293T cells (Fig. 4B). The binding sites between wild type (WT)/mutant (Mut) circTUBGCP3 3’UTR and miR-885-3p can be indicated in Fig. 4C. We found that mR-885-3p mimics could lower the luciferase activities of WT circTUBGCP3 3’UTR rather than its Mut type as compared with the miR-NC in A549 and SPC-A1 cells (Fig. 4D). RNA Immunoprecipitation (RIP) assay was performed to investigate the binding of circTUBGCP3 with Ago2-miR-885-3p complex. The RNA pulled down from Ago2 protein was used to measure the enrichment levels of endogenous circTUBGCP3 and miR-885-3p in A549 and SPC-A1 cells by RT-qPCR, which indicated that circTUBGCP3 and miR-885-3p harbored higher enrichment levels in Ago2 pellet in comparison with those in the input control (Fig. 4E). Ectopic expression of circTUBGCP3 lowered the expression of miR-885-3p (Fig. 4F), but miR-885-3p mimic exerted no effect on circTUBGCP3 expression in A549 and SPC-A1 (Additional file 1: Fig. S3). FISH showed that circTUBGCP3 was co-localized with miR-885-3p in the cytoplasm of A549 cells (Fig. 4G).

In addition, RT-qPCR showed that the expression of miR-885-3p was decreased and harbored a negative correlation with circTUBGCP3 expression in LAC tissues (Fig. 4H). The downregulation of miR-885-3p was further validated in TCGA cohorts (Fig. 4I). Low expression of miR-885-3p was associated with pathological stage (*P* = 0.006) and lymph node metastasis (*P* = 0.001, Additional file 1: Table S2). The cases with low-miR-885-3p expression harbored a worse survival as compared with those with high-miR-885-3p expression (Fig. 4I).

### MiR-885-3p reversed circTUBGCP3-caused cell growth and wnt10b signaling activation

**Fig. 5:**
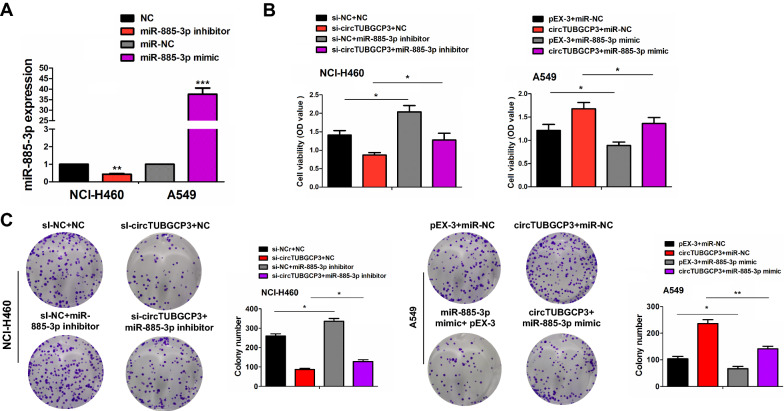
miR-885-3p reversed circTUBGCP3-induced tumor growth. **A** qPCR analysis of the efficiency of miR-885-3p inhibitor in NCI-H460 cells or its mimics in A549 cells. **B** MTT analysis of the cell viability after the co-transfection with miR-885-3p inhibitor and si-circTUBGCP3 in NCI-H460 cells or miR-885-3p mimics and circTUBGCP3 in A549 cells. **C** Colony formation analysis of the colony formation capabilities after the co-transfection with miR-885-3p inhibitor and si-circTUBGCP3 in NCI-H460 cells or miR-885-3p mimics and circTUBGCP3 in A549 cells

The efficiencies of miR-885-3p inhibitor in NCI-H460 cells or miR-885-3p mimics in A549 cells were respectively determined by RT-qPCR analysis (Fig. 5A). We found that miR-885-3p inhibitor promoted the cell proliferation and colony formation and attenuated the tumor-suppressive effect induced by circTUBGCP3 silencing in NCI-H460 cells, while miR-885-3p mimics displayed the opposite effects in A549 cells (Fig. 5B, C).

**Fig. 6:**
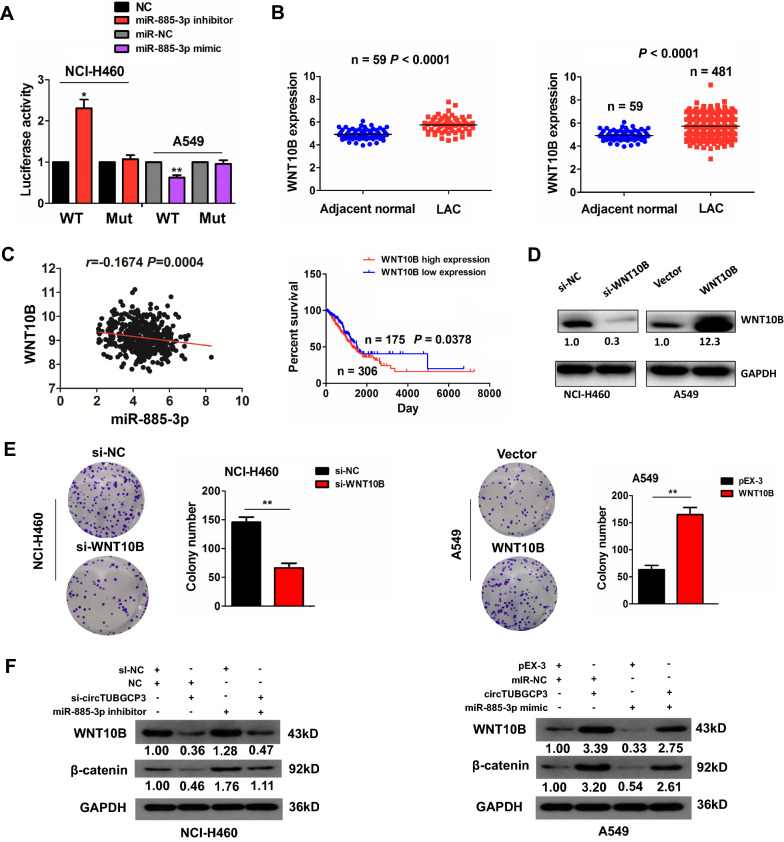
miR-885-3p reversed circTUBGCP3-induced wnt10b signaling activation. **A** The luciferase activity of WT or Mut Wnt10B 3’UTR after the treatment with miR-885-3p inhibitor in NCI-H460 cells or its mimic in A549 cells. **B** TCGA validation of the upregulation of Wnt10B in 59 paired and 481 unpaired LAC tissue samples. **C** TCGA cohort indicated the negative correlation of miR-885-3p with Wnt10B in LAC tissues, and Kaplan Meier analysis of the association of high or low Wnt10B expression with overall survival in LAC patients. **D** Western blot analysis of the efficiencies of si-wnt10b in HCI-460 cells or wnt10B plasmids in A549 cells. **E** The effects of wnt10b knockdown or overexpression on cell colony formation. **F** Western blot analysis of the activity of Wnt10B/β-catenin signaling after the co-transfection with miR-885-3p inhibitor and si-circTUBGCP3 in NCI-H460 cells or miR-885-3p mimics and circTUBGCP3 in A549 cells. Data are the means ± SEM of three experiments. *P < 0.05, **P < 0.01

According to the prediction scores by TargetScan7.1, the binding sites between WT wnt10b 3’UTR and miR-885-3p could be indicated (Additional file 1: Fig. S4). MiR-885-3p inhibitor increased the luciferase activity of WT wnt10b 3’UTR in NCI-460 cells, but mR-885-3p mimics exhibited the opposite effects in A549 cells (Fig. 6A). The upregulation of wnt10b was confirmed in 59 paired and 481 unpaired LAC tissues (Fig. 6B), and possessed a negative correlation with miR-885-3p expression in LAC tissues (Fig. 6C). The elevated expression of wnt10b was associated with gender (*P* = 0.002) and lymph node metastasis (*P* = 0.023) (Additional file 1: Table S3). The cases with wnt10b high expression had a worse survival as compared with those with wnt10b low expression (Fig. 6C). The efficiencies of si-wnt10B in NCI-H460 cells or wnt10B plasmids in A549 cells were measured by Western blot (Fig. 6D). Knockdown of wnt10B repressed cell colony formation, but restored expression of wnt10B promoted this effect (Fig. 6E). Moreover, mR-885-3p inhibitor upregulated Wnt10b and β-catenin expression and counteracted circTUBGCP3 knockdown-induced inhibitory effects on Wnt10b/β-catenin signaling activation in NCI-H460 cells, but miR-885-3p mimics harbored the opposite effects in A549 cells (Fig. 6F).

### Knockdown of circTUBGCP3 inhibited the tumorigenesis of LAC

**Fig. 7:**
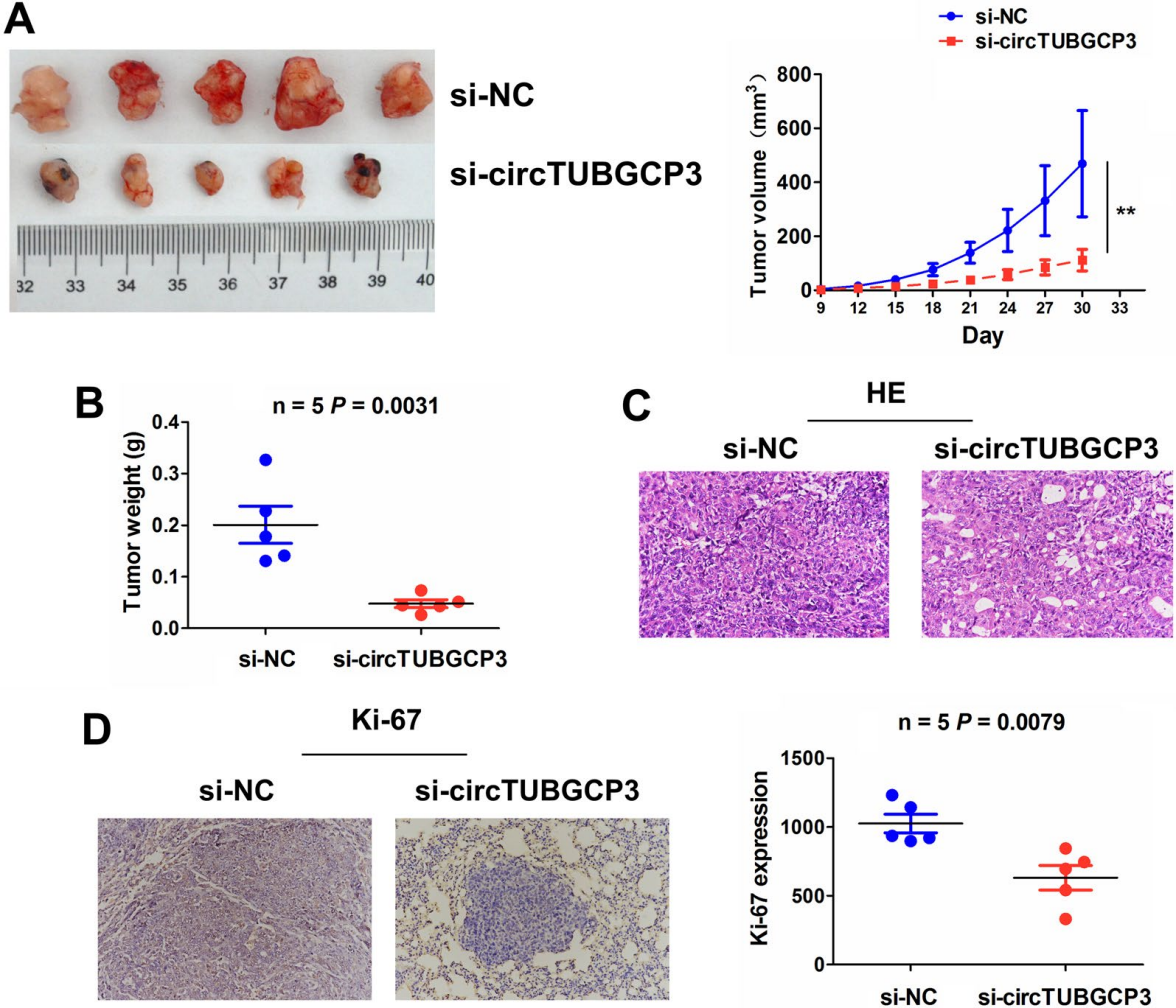
Knockdown of circTUBGCP3 inhibited the tumorigenesis of LAC. **A** Comparing the tumor size of xenograft tumors between si-circTUBGCP3 and si-NC transfected NCI-H460 cells, and a growth curve analysis of the tumor growth between these two groups. **B** Comparing the tumor weight of xenograft tumors between si-circTUBGCP3 and si-NC groups. **C** HE analysis of the cell proliferating number between si-circTUBGCP3 and si-NC groups. **D** IHC analysis of the levels of Ki-67 between si-circTUBGCP3 and si-NC groups

To determine whether circTUBGCP3 influenced in vivo tumorigenesis of LAC, we established a xenograft tumor model in nude mice with lentivirus-mediated si- circTUBGCP3 or si-NC stably-transfected NC-H460 cells, and then subcutaneously injected these cells into the flank of nude mice. After an observation for 30 days, we found that, the volumes of the xenograft tumors induced by si-circTUBGCP3 transfected NC-H460 cells were markedly smaller than those induced by si-NC, and the growth curve demonstrated that, the tumors in si-circTUBGCP3 group grew slower (Fig. 7A). The tumor weight was obviously slighter in si-circTUBGCP3 group than that in si-NC group (Fig. 7B). HE staining indicated a decreased number of proliferating tumor cells in si-circTUBGCP3 group as compared with the si-NC group (Fig. 7C). IHC analysis showed that the levels of Ki-67 were significantly reduced in si-circTUBGCP3 group as compared with the si-NC group (Fig. 7D).

## Discussion

A variety of studies have shown that circRNAs such as hsa_circ_0001946 [16] can be used as diagnostic markers for NSCLC. Herein, we identified a differentially-expressed circTUBGCP3 between LAC and adjacent normal tissues, and uncovered that circTUBGCP3 as an independent prognostic factor was associated with pathological stage and poor survival in LAC. TNM stage is of significance to cancer prognosis [17]. We found that upregulation of circTUBGCP3 indicated an unfavorable prognosis in early stage LAC.

It has been shown that circ_100146 [18] and circFOXM1 [19] act as oncogenic factors to promote NSCLC progression, whereas circ_0078767 [20] and circPTPRA [12] as tumor suppressors control epithelial-mesenchymal transition (EMT) and NSCLC metastasis. Herein, we found that ectopic expression of circTUBGCP3 promoted the tumorigenesis of LAC cells, while silencing of circTUBGCP3 attenuated these effects in vitro and in vivo. Our findings indicated circTUBGCP3 might be an oncogenic factor in LAC.

Accumulating data indicate that circRNAs can sponge miRNAs to participate in the progression of NSCLC. CircFGFR1 [21]and circZFR [22] sponge miR-381-3p or miR-101-3p to augment the progression and anti-PD-1 resistance in NSCLC, but hsa_circ_0008305 [23] and circ_0001649 [24]sponge miR-429/-200b-3p or miR-331-3p/-338-5p to depress TGF-β-induced EMT and metastasis in NSCLC. Herein, we found that circTUBGCP3 could sponge miR-885-3p to favor the growth of LAC. Some studies have indicated that miR-885 suppresses the angiogenesis of colon cancer [25], but promotes the invasion of gastric cancer [26]. HOXB-AS1/miR-885-3p/HOXB2 axis boosts the bio-behaviors of glioblastoma [27]. We here found that downregulation of miR-885-3p was associated with pathological stage, lymph node metastasis and poor survival in LAC, and miR-885-3p repressed the proliferation and colony formation of LAC cells and antagonized circTUBGCP3-induced tumor proliferation. These results implied that circTUBGCP3 might sponge miR-885-3p to promote the tumorigenesis of LAC.

**Fig. 8:**
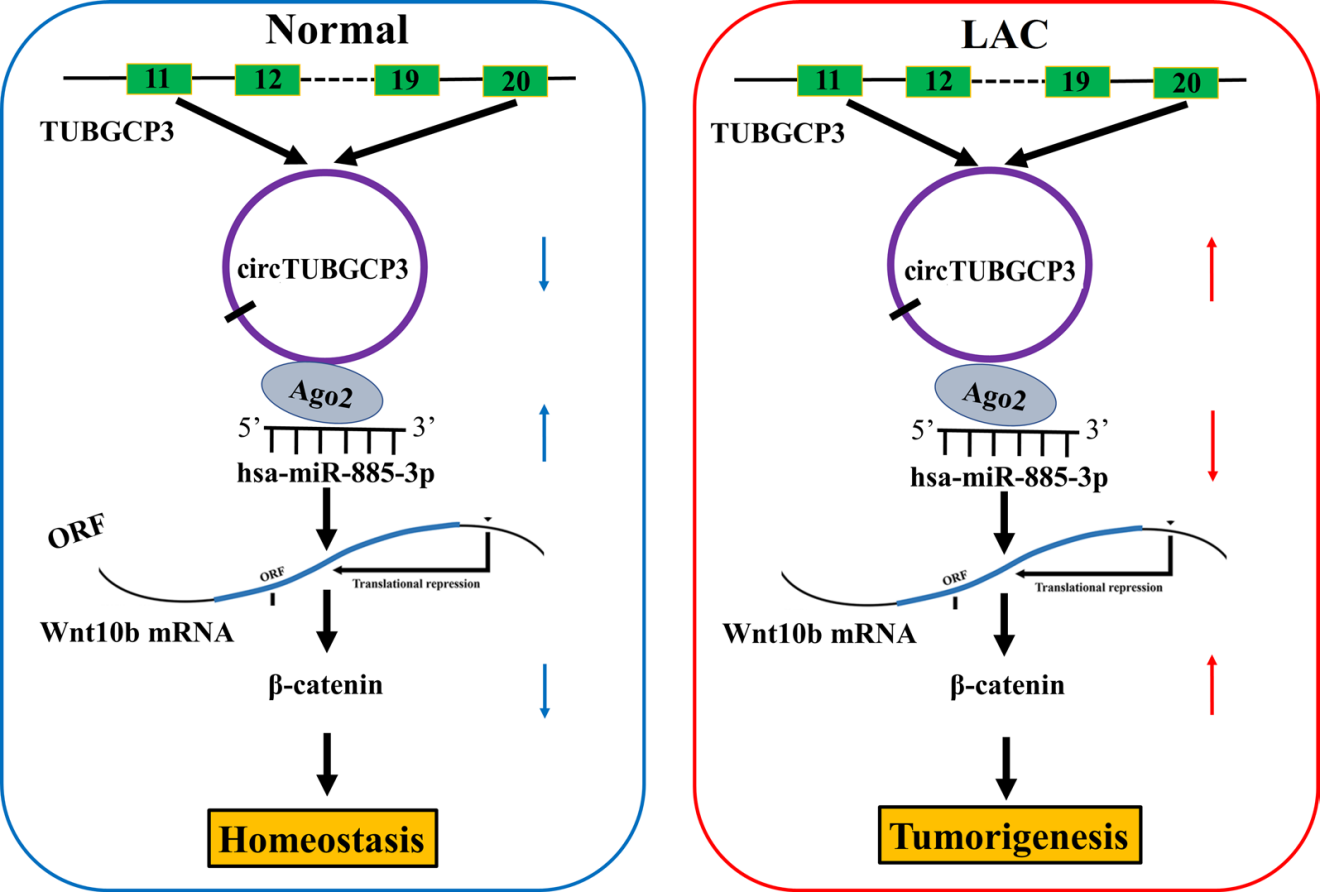
Schematic representation of the proposed mechanism of circTUBGCP3 in LAC. CircTUBGCP3 acted as a sponge of miR-885-3p to activate the Wnt10b/β-catenin signaling, leading to the tumorigenesis of LAC

We then identified Wnt10b as a direct target of miR-885-3p in LAC. It has been shown that high expression of Wnt10b is associated with the survival and metastases in breast cancer [28] and prostate cancer [29]. Wnt10b/β-catenin signaling facilitates HMGA2 expression and cell proliferation in breast cancer [30], and miR-148a inhibits the invasion of colorectal cancer by targeting Wnt10b/β-catenin signaling [31]. We herein found that miR-885-3p reduced cell growth and attenuated circTUBGCP3-induced cell growth by targeting Wnt10b/β-catenin signaling. Upregulation of Wnt10b was associated with gender, pathological stage and poor survival in LAC. CircTUBGCP3 facilitated the tumorigenesis of LAC cells by sponging miR-885-3p and activating Wnt10b/β-catenin signaling (Fig. 8).

In conclusion, our findings demonstrated that circTUBGCP3 facilitated the tumorigenesis of LAC by sponging miR-885-3p and activating Wnt10b/β-catenin signaling. Our findings might provide a novel insight into the detection of LAC.

## Supplementary Information

**Additional file 1: Figure S1.**(A) The circRNA profiling of the differentially expressed circRNAs between LAC andadjacent normal tissues. (B) The locus and origination of circTUBGCP3. (C)Kaplan-Meieranalysis of the association of high or low circTUBGCP3 expressionwith overall survival in late-stage cases. **Figure S2.** Thebinding sites between WT or Mut circTUBGCP3 3’UTR and 5 miRNAs. **Figure S3. **qPCR analysis of the expression levels ofcircTUBGCP3 after the transfection with miR-885-3p mimics in A549 and SPC-A1.** Figure S4.** Thebinding sites between WT or Mut Wnt10B 3’UTR and miR-885-3p. **Table S1. **The primer sequences. **Table S2.** Theassociation of miR-885-3p expression with clinicopathological characteristics inLAC patients. **Table S3.** The association of Wnt10b expression with clinicopathological characteristics in LACpatients.

## Data Availability

The datasets used during the current study are available from the corresponding author on reasonable request.
