# Supplementary material for: CircTUBGCP3 facilitates the tumorigenesis of lung adenocarcinoma by sponging miR-885-3p
Source: Cancer Cell Int. 2021 Dec 6;21:651. doi: 10.1186/s12935-021-02356-2 (PMC8647460; doi:10.1186/s12935-021-02356-2)
Supplement: Supplementary file 1 — Additional file 1: Figure S1.(A) The circRNA profiling of the differentially expressed circRNAs between LAC andadjacent normal tissues. (B) The locus and origination of circTUBGCP3. (C)Kaplan-Meieranalysis of the association of high or low circTUBGCP3 expressionwith overall survival in late-stage cases. Figure S2. Thebinding sites between WT or Mut circTUBGCP3 3’UTR and 5 miRNAs. Figure S3. qPCR analysis of the expression levels ofcircTUBGCP3 after the transfection with miR-885-3p mimics in A549 and SPC-A1. Figure S4. Thebinding sites between WT or Mut Wnt10B 3’UTR and miR-885-3p. Table S1. The primer sequences. Table S2. Theassociation of miR-885-3p expression with clinicopathological characteristics inLAC patients. Table S3. The association of Wnt10b expression with clinicopathological characteristics in LACpatients. [file 12935_2021_2356_MOESM1_ESM.docx]

**Supplementary Figures.**

**Figure S1 (A)** The circRNA profiling of the differentially expressed circRNAs between LAC and adjacent normal tissues. **(B)** The locus and origination of circTUBGCP3. **(C)** Kaplan-Meier analysis of the association of high or low circTUBGCP3 expression with overall survival in late-stage cases.

**Figure S2** The binding sites between WT or Mut circTUBGCP3 3’UTR and 5 miRNAs.

**Figure S3** qPCR analysis of the expression levels of circTUBGCP3 after the transfection with miR-885-3p mimics in A549 and SPC-A1.

**Figure S4** The binding sites between WT or Mut Wnt10B 3’UTR and miR-885-3p.

**Supplementary Tables**

**Table S1** The primer sequences

| **Gene name** | **Primer sequence** | Annealing temperature (℃) | Product length (bp) |
| --- | --- | --- | --- |
| GAPDH（Human） | F:5' ACAACTTTGGTATCGTGGAAGG3'  R:5’ GCCATCACGCCACAGTTTC3’ | 60 | 101 |
| circTUBGCP3 | F:5’ GGATCACATCATTGCTGCAC3’  R:5’ TGCCACAGTCGATCTGTTTT3’ | 60 | 200 |
| miR-885-3p | F: 5’ CGCGGTATGGCACTGGTAGA 3’ | 60 |  |
|  | R:5’ AGTGCAGGGTCCGAGGTATTC3’ |  |  |
| Wnt10B | F: 5’TGAGAGACCAATACATGAGGACA3’  R:5’ CGGTTGTGGGTATCAATGAAGA 3’ | 60 | 204 |
| EIF4A3 | F: 5’ GGGGCATCTACGCTTACGG3’  R:5’ GCGATGACATCTCTCCCTTTGA3’ | 60 | 88 |
| TUBGCP3 | F: 5’ TGCTACGTTATTTGCTCAGGC3’  R:5’ TGGGCACTCCGATCTTGGTA3’ | 60 | 105 |

**Table S2** The association of miR-885-3p expression with clinicopathological characteristics in LAC patients

| Variables | Cases  (n) | miR-885-3p | | *P* value |
| --- | --- | --- | --- | --- |
|  |  | High | Low |  |
| Total | 419 | 348 | 71 |  |
| *Age (years)* |  |  |  |  |
| ≥60 | 302 | 253 | 49 |  |
| <60 | 117 | 95 | 22 | 0.562 |
| *Gender* |  |  |  |  |
| Male | 194 | 167 | 27 |  |
| Female | 225 | 181 | 44 | 0.151 |
| *Pathological stage* |  |  |  |  |
| Ⅰ/Ⅱ | 332 | 285 | 47 |  |
| Ⅲ/Ⅳ | 87 | 63 | 24 | 0.006* |
| *T stage* |  |  |  |  |
| T1/T2 | 363 | 304 | 59 |  |
| T3/T4 | 56 | 44 | 12 | 0.341 |
| *N stage* |  |  |  |  |
| Negative | 277 | 242 | 35 |  |
| Positive | 142 | 106 | 36 | 0.001* |
| *M stage* |  |  |  |  |
| Negative | 261 | 215 | 46 |  |
| Positive | 158 | 133 | 25 | 0.688 |

**Table S3** The association of Wnt10b expression with clinicopathological characteristics in LAC patients

| Variables | Cases  (n) | Wnt10b | | *P* value |
| --- | --- | --- | --- | --- |
|  |  | High | Low |  |
| Total | 481 | 306 | 175 |  |
| *Age (years)* |  |  |  |  |
| ≥60 | 350 | 216 | 134 |  |
| <60 | 131 | 90 | 41 | 0.167 |
| *Gender* |  |  |  |  |
| Male | 220 | 156 | 64 |  |
| Female | 261 | 150 | 111 | 0.002* |
| *Pathological stage* |  |  |  |  |
| Ⅰ/Ⅱ | 378 | 236 | 142 |  |
| Ⅲ/Ⅳ | 103 | 70 | 33 | 0.355 |
| *T stage* |  |  |  |  |
| T1/T2 | 417 | 263 | 154 |  |
| T3/T4 | 64 | 43 | 21 | 0.578 |
| *N stage* |  |  |  |  |
| Negative | 312 | 187 | 125 |  |
| Positive | 169 | 119 | 50 | 0.023* |
| *M stage* |  |  |  |  |
| Negative | 318 | 208 | 110 |  |
| Positive | 163 | 98 | 65 | 0.271 |
